# Acrylate-assisted fractal nanostructured polymer dispersed liquid crystal droplet based vibrant colored smart-windows†

**DOI:** 10.1039/c9ra00729f

**Published:** 2019-04-24

**Authors:** Sunil Kumar, Hyeryeon Hong, Woosuk Choi, Imtisal Akhtar, Malik Abdul Rehman, Yongho Seo

**Affiliations:** Graphene Research Institute and HMC, Sejong University Seoul 05006 South Korea yseo@sejong.ac.kr; Department of Nanotechnology and Advanced Materials Engineering, Sejong University Seoul 05006 South Korea

## Abstract

We have studied liquid crystals (LCs) and acrylate-assisted thiol–ene compositions to synthesize dye based colorful polymer dispersed liquid crystals (PDLCs) without using a photo-initiator for smart-windows applications. A typical PDLC mixture was prepared by mixing LCs with UV-curable monomers, which included triethylene glycol diacrylate (TEGDA), trimethylolpropane diallyl ether (TMPDE, di-functional ene monomer), trimethylolpropane tris(3-mercaptopropionate) (TMPTMP, a thiol as a cross-linker), and a dichroic dye. The ratios of the TMPDE/TMPTMP and the LCs/TEGDA showed significant effects in altering the properties of the UV-cured PDLCs. During the curing process, the monomers polymerize and led to the encapsulation of the LCs in the form of interesting fractal nanostructures by a polymerization induced phase separation process. The switching time, electro-optical properties, power consumption, and ageing of the fabricated PDLCs were investigated. It was possible to achieve a 70–80% contrast (Δ*T*) at a voltage difference of ∼70 V with a fast switching time (*τ*) as low as < 20 milliseconds (ms) and low power consumption. These PDLCs had a low threshold voltage that ranged between 10 and 20 V. The sustainability of the fabricated UV-cured PDLCs was analyzed for up to 90 days, and the PDLCs were observed to be stable.

## Abstract 2

Vibrant colored smart-windows were fabricated based on acrylate-assisted fractal nanostructured polymer dispersed liquid crystals.

## Graphical abstract

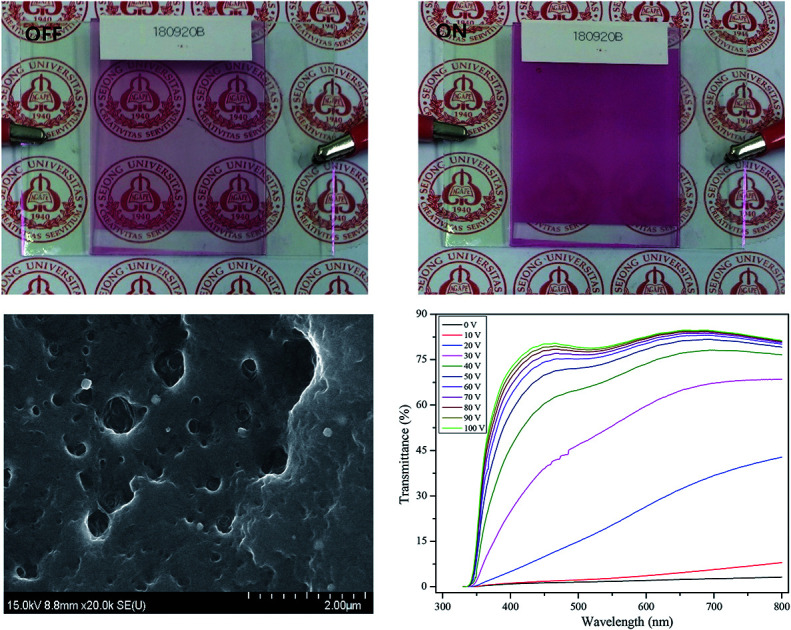

## Introduction

Energy conservation and global warming are two major issues closely interrelated with each other. The whole world is concerned about adopting different methods to conserve energy, so it can be utilized where it is ultimately needed. One such approach for energy conservation is the use of smart or switchable glass windows.^1^ These not only let light pass through or block it but also save the architectural cost of using simple glass or wooden/curtain based windows and reduce excess heating or cooling, which in turn makes a contribution towards minimizing global warming with the use of switchable glazing.^2^ Smart windows^3,4^ are mainly based on polymer dispersed liquid crystals (PDLCs), electrochromic materials^5^ and suspended particle devices (SPD).^1,6,7^ Because these windows have two different states, which are transparency and opacity, they give the user effortless control over the surrounding interruptions needed for privacy. These smart windows are becoming popular especially in commercial office spaces, because they exhibit an aesthetic look, incorporate different designs and colorful dyes, and allow light to pass through them, which results in saving energy. Besides these, PDLCs have applications in holographic grating,^8^ light diffusion,^9,10^ display devices,^11^ and electrochromic devices.^12^

When a voltage is applied to a PDLC, the LC domains align themselves in a parallel direction along the applied field, which leads to an ON state (transparent), and zero voltage causes the OFF state (opaque). PDLCs based smart windows have encapsulated LCs droplets formed by the polymerization induced phase separation (PIPS) process when the PDLC mixture, which is sandwiched between ITO glasses by the capillary action, is cured using a suitable technique, which is generally UV-curing. The switching mechanism of PDLCs is exploited by means of the birefringence (Δ*n* = *n*_e_ − *n*_o_, where *n*_e_ is extraordinary and *n*_o_ is ordinary refractive index), which must be high to give a good contrast ratio (ratio of ON state transmittance *T*_ON_ to OFF state transmittance *T*_OFF_). Another condition for good contrast is the matching of the refractive index of polymer (*n*_p_) used in PDLCs with the ordinary refractive index of LCs *i.e. n*_o_ ≈ *n*_p_. Most of the PDLCs published in recent times are based on commercial polymers, such as NOA65 and NOA68, or other costly polymers and LCs like 5-CB (Δ*n* = 0.191), BL038 (Δ*n* = 0.272), SLC 1717 (Δ*n* = 0.201), and E-7 (Δ*n* = 0.222 and 0.225). However, there are other monomers that can be used as a cross-linker to reinforce the solidity of the polymer. There is another class of PDLCs based on thiol–ene systems. The PDLCs mixture based on thiol–ene click chemistry leads to the good dispersibility between the constituents resulting in the formation of uniform polymerized networks.^13,14^ Such thiol–ene based systems, in addition to smart windows, can also be used in holographic PDLCs reflection grating.^8^ The thiol–ene based polymerizations are not inhibited by oxygen, which eliminates the need to add photoinitiators.^15^ Recently, few attempts have been made to fabricate PDLCs based on such systems. Zhang *et al.* studied the effect of a cross-linker (PEGDA) and diluents (hydroxypropyl methacrylate (HPMA)) and lauryl methacrylate (LMA) on the morphology and properties of thiol–ene based PDLCs.^16^ Although there are some reports of non-dye PDLCs based thiol–ene systems,^17–21^ the fabrication of colorful PDLCs with thiol–ene systems are still a challenge. Shi *et al.* have attempted the dye-doped PDLCs with thiol–ene systems with good switching and low driving voltage, however, the colored appearance was not observed in the OFF state.^22^ The colorful PDLCs can be obtained by adding various types of dyes, such as azo as well as anthraquinone. It has been observed that the contrast ratio of a PDLC is improved with the addition of dye.^23,24^ When a dye (guest) is added to the LCs (host), it gives rise to a guest–host (G–H) configuration, and it leads to the formation of guest–host PDLCs (GHPDLCs).^25^ In such systems, the orientation of dye molecules is controlled by the LCs in an applied electric field, but the dye molecules in a polymer matrix are not affected by the field. There are many reports on dye based PDLCs,^26–28^ but almost none of them presented a colorful appearance of the PDLCs cells in the OFF state. However, there are some exceptions other than thiol–ene based PDLCs, but the aesthetic appearance in these PDLCs is missing.^24,26^

To the best of our knowledge, there are few or no studies on vibrant colored PDLCs having thiol–ene systems. In this study, we optimized the acrylate assisted thiol–ene systems, which was based on GH configuration, such as dye-based colored PDLCs, without a photo-initiator and having a colorful appearance in the OFF state to obtain the PDLCs with a vibrant aesthetic appearance.

## Materials and methods

### Materials

The fabricated PDLC mixtures were based on commercial LCs (C7, Qingdao Intermodal Trading Ltd, China, *n*_o_ = 1.52, *n*_e_ = 1.73) consisting of 4-pentylphenyl 4-propylbenzoate (63 wt%) and 4 *N*-pentylbiphenyl (37 wt%). The monomers used were triethylene glycol diacrylate (TEGDA) (*n* = 1.461), trimethylolpropane diallyl ether (TMPDE) (*n* = 1.458), trimethylolpropane tris (3-mercaptopropionate) (TMPTMP) (*n* = 1.518) (all from Sigma Aldrich, USA) [Chemical structures in Fig. S3, ESI†], and NOA65 (Norland Products Inc., USA). The colored PDLCs were obtained by introducing red (AR1) or blue (AB4) dichroic dyes (Nematel GmbH & Co. KG, Germany). The red dye, AR1, is an azo dye which is also known as Amido Naphthol Red G or azophloxine. Both dyes are dichroic dyes which generally have rod-like shapes. According to the data sheet, the maximum absorbance wavelengths (*λ*_max_) are ∼554 nm and ∼641 nm for AR1 red dye and Blue AB4 dye, respectively. These kinds of dyes are mainly used for dyeing fabrics and foods.^29^ These dyes absorb light polarized parallel to its elongated direction more strongly than that in perpendicular direction.^30^ Thus, the switching behavior of PDLC has been improved by adding dichroic dyes, because it tends to align with LC.^23^ The mixtures were stirred using an Ultra Turrax homogenizer (IKA Korea Ltd., South Korea). The ITO-coated glasses (size 50 mm × 50 mm, *Ω* ∼7 ohm per square, Wooyang GMS, South Korea) used to fabricate cells were separated using a 25 μm PET film as a spacer. These cells were irradiated using UV-light (Hg lamp, power 1 kW) for 30 minutes at a distance of 23 cm to cure the LCs and the monomers mixture. The LCs droplets formation were determined using a field emission scanning electron microscope (FESEM, SU8010 Hitachi, Japan). The studies of the electro-optical and switching response of the fabricated PDLCs were investigated using a UV-visible spectroscopy (Cary 5000, Varian, USA) and an oscilloscope (Tektronix, USA) at various voltages using an AC voltage driver with a supply voltage between 0–100 V.

### Method

In a typical method, LCs, TEGDA as an acrylate monomer, TMPDE as a di-functional ene monomer, TMPTMP as a cross-linker thiol, and red (or blue) dye in the case of color PDLCs, were mixed together, in desired wt%, in a vial for 30 minutes at room temperature using a homogenizer at ∼20 000 rpm. The homogenous mixture was sandwiched between the ITO glasses by capillary action and cured by irradiating UV-light (intensity ∼95.4 mW cm^−2^) for 30 minutes. During the curing process, the monomers polymerized and led to the encapsulation of the LCs in the form of droplets by the PIPS process. In this process the phase separation and polymerization occur at the same time so that LCs are segregated from the polymerized matrix forming droplets. The cells were allowed to cool at room temperature before making further analysis. Different compositions of the fabricated PDLCs are summarized in Table 1. Among these compositions, further studies were carried out with best compositions of LCs and monomers in non-dye and red dye (0.7 wt%) based PDLCs. For comparison, the NOA65 based PDLCs were also fabricated under similar conditions and concentrations of LCs, monomers and red or blue dyes (0.7 wt%). Besides these, the blue dye based PDLCs were fabricated for comparison with the red dye. The experimental results for blue dye are described in ESI, Section 1 and 2,† which were not much different from results for the red dye. To summarize, various compositions of LCs, monomers and dyes based PDLCs were fabricated, and the selected PDLCs were further studied.

**Table 1:** Samples list with different ratio of LCs/monomers/AR1 red dye to optimize suitable PDLCs composition

S. no.	LCs (wt%)	TEGDA (wt%)	TMPDE (wt%)	TMPTMP (wt%)	Red dye (wt%)
1	55.0	45.0	0.0	0.0	0.0
2	55.0	0.0	22.5	22.5	0.0
3	52.5	30.0	9.25	9.25	0.0
4	54.0	27.0	9.5	9.5	0.0
5	56.0	25.0	9.5	9.5	0.0
6	57.5	23.5	9.5	9.5	0.0
7	61.5	20.5	9.0	9.0	0.0
8	55.0	27.0	7.2	10.8	0.0
9	55.0	27.0	9.0	9.0	0.0
10	55.0	27.0	10.8	7.2	0.0
11	55.0	27.0	12.0	6.0	0.0
12	54.3	27.0	10.8	7.2	0.7
13	54.3	27.0	9.0	9.0	0.7
14	54.3	27.0	8.0	10.0	0.7
15	54.3	27.0	7.2	10.8	0.7
16	54.0	27.0	8.0	10.0	1.0
17	54.5	27.0	8.0	10.0	0.5

### LCs/TEGDA ratios optimization for non-dye PDLCs

The fabrication of PDLCs using LCs with TEGDA only or LCs with TMPDE/TMPTMP compositions have good OFF state transmittance but low ON state transmittance as indicated in Table S1 and Fig. S2(A–B1) [ESI†]. However, LCs/TEGDA/TMPDE/TMPTMP compositions, at appropriate ratios, exhibited better transmittance change, so the ratios of these components were optimized to obtain suitable composition. Hence, to start with, the LCs/TEGDA ratio has been optimized while keeping the TMPDE/TMPTMP ratio constant. The associated parameters are summarized in Table 2. Among these parameters, Δ*T* (%) (*T*_ON_ − *T*_OFF_), which is the difference between the ON/OFF state transmittance (%), is the most important. The other parameters include power consumption (W m^−2^) and switching time *τ* (ms).

**Table 2:** Different parameters of non-dye PDLCs at different LCs/TEGDA ratios

S. no.	LCs/TEGDA ratio (wt%)	Δ*T* (%)	Power (W m^−2^)	Switching time (ms)	
				Rise	Fall
1	1.75 : 1	69	5.8	0.38	54
2	2 : 1	77	5.9	0.20	36
3	2.25 : 1	70	6.4	0.32	88
4	2.5 : 1	69	6.1	0.75	111
5	3 : 1	53	6.8	1.30	121

It can be seen clearly that a ∼2 : 1 ratio gives the best set of parameters, which includes Δ*T* ∼77%, power ∼5.9 W m^−2^ and rising/falling times (*τ*) ∼0.20/36 ms. As the ratio is increased, Δ*T* decreases, and the power and *τ* increase with few exceptions. The variation trend of the parameters of the PDLCs at different LCs/TEGDA ratios are shown as a histogram in Fig. S1(A) [ESI†].

### TMPDE/TMPTMP ratios optimization for non-dye PDLCs

Owing to the thiol–ene clickable reaction mechanism,^31^ the TMPDE/TMPTMP ratio optimization is an important part of a PDLCs. After optimizing suitable LCs/TEGDA ratio, PDLCs compositions with different TMPDE/TMPTMP ratios (wt%), which kept the LCs and TEGDA at ∼2 : 1, have been studied, and the associated parameters are summarized in Table 3.

**Table 3:** Different parameters of PDLCs at different TMPDE/TMPTMP ratios

S. no.	TMPDE/TMPTMP ratio (wt%)	Δ*T* (%)	Power (W m^−2^)	Switching time (ms)	
				Rise	Fall
1	1 : 1.5	52	8.2	0.26	20
2	1 : 1	77	5.9	0.20	36
3	1.5 : 1	72	7.3	0.36	49
4	2 : 1	43	10.2	4.50	19

From Table 3, it is observed that TMPDE/TMPTMP ratio alters the parameters of the PDLCs, and at a 1 : 1 ratio the PDLCs have high Δ*T* (77%), the lowest power (∼5.9 W m^−2^), and good *τ* ∼ 36 ms. It was observed that the uniformity of the PDLCs deteriorates, and Δ*T* is low when the TMPDE/TMPTMP ratio is higher or lower (other than 1 : 1), which may be attributed to the mismatch of the TMPDE/TMPTMP ratio, because the polymerization is affected by their ratio, which will be discussed in the polymerization mechanism section. Hence, at a fixed LCs/TEGDA ratio (2 : 1), the PDLCs at a 1 : 1 ratio of the TMPDE/TMPTMP offered the better set of parameters. The variation of parameters of the PDLCs at different TMPDE/TMPTMP ratios are shown in Fig. S1(B) [ESI†].

### Colored PDLCs optimization

The vibrant red colored PDLCs were fabricated by optimizing concentration of TMPDE/TMPTMP ratio while keeping the LCs/TEGDA ratio at ∼2 : 1 and red dye at ∼0.7 wt%. The dependence of the TMPDE/TMPTMP ratio are summarized in Table 4, and the variation trend is shown in Fig. S1(C) [ESI†]. It was found that the TMPDE/TMPTMP ratio for the optimized sample was slightly different as observed in the non-dye case. It was observed that in the colored PDLCs, the Δ*T* improved as the TMPTMP increased to 1.25 times of the TMPDE, and then again it was found slightly reduced when it was raised to 1.5 times, but it had good power and switching response time characteristics. The slight difference in the parameters as compared to non-dye counterpart was attributed to the dye addition. As a result, it was concluded that the best set of parameters were obtained at 1 : 1 and 1 : 1.25 TMPDE/TMPTMP ratios for the non-dye and colored PDLCs, respectively, while keeping the LCs/TEGDA at 2 : 1.

**Table 4:** Parameters of red colored PDLCs at different TMPDE/TMPTMP ratios

S. no.	TMPDE/TMPTMP ratio (wt%)	Δ*T* (%)	Power (W m^−2^)	Switching time (ms)	
				Rise	Fall
1	1.5 : 1	41	10.9	0.44	13
2	1 : 1	52	9.7	0.28	16
3	1 : 1.25	73	6.4	0.30	17
4	1 : 1.5	69	6.1	0.42	28

The best LCs and monomers compositions *i.e.* LCs (∼55 wt%), TEGDA (∼27), TMPDE (∼8 wt%) and TMPTMP (∼10 wt%) optimized at intermediate red dye concentration 0.7 wt%, was also tested at various dye concentrations from 0.5 wt% to 1 wt%, respectively. The associated parameters are shown in Table 5. Though the powers and switching times have minute differences, the PDLCs at ∼1 wt% concentration have low Δ*T* as compared to low dye concentration 0.7 wt%. The sample with 0.7 wt% dye concentration has slightly low Δ*T* as compared to 0.5 wt%, which may be ascribed to absorption of light by dye molecules. Earlier reports also have similar trend at higher dye concentrations.^32,33^ Also, the PDLCs with 1 wt% dye have more red colored appearance in the ON state, but good opacity in OFF state. Similarly, at lower dye concentration (∼0.5 wt%) the ON state is comparatively transparent, but OFF state shows fainted red color as evidenced from Fig. S2(G and H) [ESI†]. Hence, further investigations were carried with 0.7 wt% concentration, which was compared with best non-dye PDLCs.

**Table 5:** Parameters of colored PDLCs at different dye concentrations

S. no.	Red dye (wt%)	Δ*T* (%)	Power (W m^−2^)	Switching time (ms)	
				Rise	Fall
1	0.5	74	6.2	0.21	25
2	0.7	73	6.4	0.30	17
3	1.0	68	6.7	0.33	14

### Polymerization mechanism in PDLCs

**Fig. 1:**
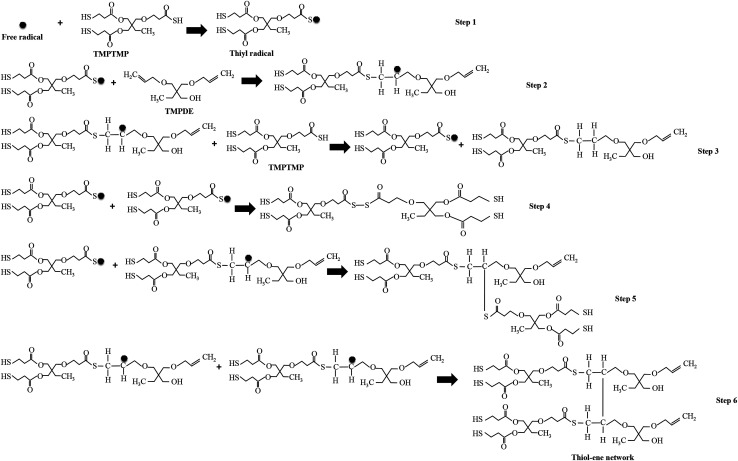
Thiol–ene reaction involved polymerization mechanism of TMPTMP/TMPDE.

The polymerization in the encapsulated PDLCs was based on thiol–ene reaction mechanism^34,35^ and acrylate homo-polymerization.^36^ In this case, the TMPTMP is thiol, the TMPDE is ene, and the TEGDA is acrylate. The thiol–ene reaction was initiated from the free radicals formed by the absorption of the UV-light without photoinitiator.^15,37^ Consequently, the abstraction of the hydrogen atom from the thiol monomer (TMPTMP) by free radicals led to the formation of thiyl radicals, which is shown as step 1 in Fig. 1. The thiyl radicals had a tendency to react with the unsaturated monomer, for instance, the ene monomer (TMPDE) had carbon–carbon double (C=C) bonds. As these radicals interacted with the ene monomer, it led to the formation of carbon-based-radicals, which is indicated in step 2. These carbon based free radicals can react with another TMPTMP, and another thiyl radical and thiol–ene chain can be generated (step 3), which can be involved in next thiol–ene reaction as it has the other double bond. The radicals can be terminated by the different reactions, which is shown in steps 4 to 6, but the resultant molecules have double bonds to be involved in further thiol–ene reactions. The above mechanism showed the simplest network formation, and the chains generated at different steps were further activated by free radicals that led to complex free radical chains and then ultimately the complex network. In particular, the molecule after step 6 had 2 double bonds, and it can be substituted for the TMPDE in the step 2 reaction. Thus, the alkyl groups were linked by repeated steps 2 and 6, which formed a thiol–ene network as a polymerization. Also, the TEGDA had 2 double bonds (Fig. S3, in ESI†) that were involved in thiol–ene reaction. In addition to the thiol–ene polymerization process, there was another polymerization that occurred at the same time *via* the acrylate like TEGDA, which led to the formation of a homo-polymerized network by acrylate–acrylate homo-polymerization^36^ along with the thiol–ene polymerized network. It gives rise to long complex chained polymerized structures^38^ as thiol–ene–acrylate network.^39^ From this type of polymerization network, fractal structures were expected due to the presence of thiol and acrylate.^35,40^

## Results and discussion

### Visual characteristics (ON/OFF states)

**Fig. 2:**
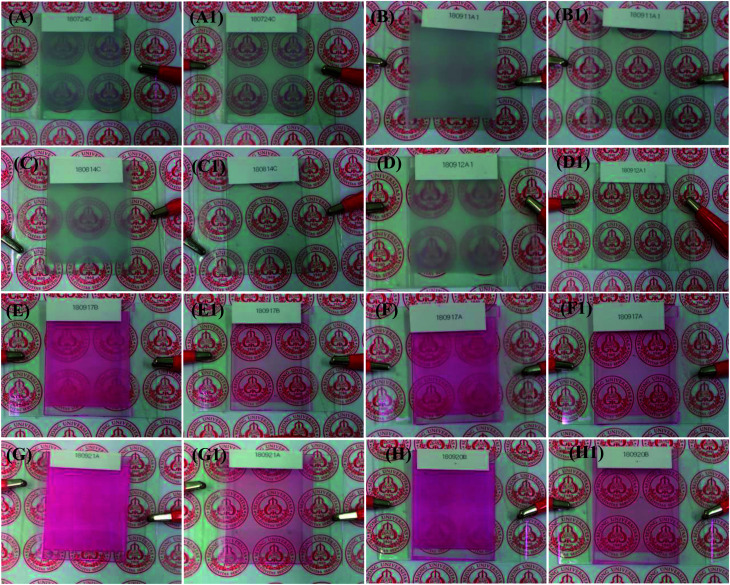
Visual characteristics: the obvious change in the ON/OFF states can be clearly seen in the photographs. The OFF/ON states of various PDLCs without dye at 0/100 *V*_ac_: (A/A1), (B/B1), (C/C1), and (D/D1), where the TMPDE/TMPTMP ratios are 2 : 1, 1.5 : 1, 1 : 1, and 1 : 1.5. Photographs in the OFF/ON states of the red colored PDLCs are shown. The TMPDE/TMPTMP ratios are 1.5 : 1, 1 : 1, 1 : 1.25, and 1 : 1.5: (E/E1), (F/F1), (G/G1), and (H/H1), respectively.

The visual images of the PDLCs ON/OFF states at 0/100 *V*_ac_ are shown in Fig. 2(A–H1). The non-dye UV-cured PDLCs were found to appear as turbid white in the OFF state, and the transparent state (ON) was seen with a clear background. The dye based PDLCs had a vibrant pinkish red color in the OFF state, and they were transparent in the ON state with some fainted pinkish red that may be ascribed to some dye molecules in polymer matrix, which were not entrapped inside the LCs droplets. It was observed that in the OFF state, the PDLCs had different transmittances, which depended on the TMPDE/TMPTMP ratios, whereas in the ON state, the background was seen clearly in all the PDLCs, which indicated high transparency.

### FE-SEM analysis

**Fig. 3:**
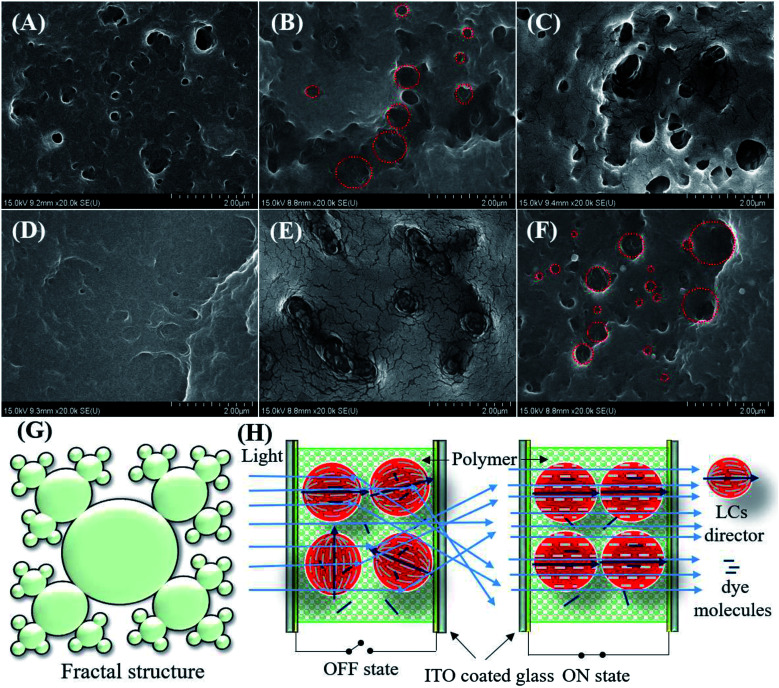
Morphological analysis: FESEM images of (A–C) non-dye, (D–F) colored PDLCs, where the TMPDE/TMPTMP ratios are (A) 1.5 : 1, (B) 1 : 1, (c) 1 : 1.5, (D) 1.5 : 1, (E) 1 : 1, and (F) 1 : 1.25, respectively. (G) Artistic impression of the fractal structured LCs droplets and (H) a schematic of the ON/OFF switching mechanism of dye based PDLCs.

The LCs droplet sizes were estimated using FE-SEM. The surface morphology of the polymer networks of the non-dye PDLCs are shown in Fig. 3(A–F). For this analysis, the ITO glasses were separated and dipped in cyclohexane for four days to remove the LCs from the fractured PDLCs. The LCs removed glasses with the polymer matrix were dried at 60 °C for 12 hours. It was observed that the size of the droplets ranged between 100–500 nm. The morphology of the pores varied with the TMPDE/TMPTMP ratio in the non-dye PDLCs at 1.5 : 1, 1 : 1, and 1 : 1.5 TMPDE/TMPTMP ratios [Fig. 3(A–C)], and in the red colored PDLCs at 1.5 : 1, 1 : 1, and 1 : 1.25 TMPDE/TMPTMP ratios [Fig. 3(D–F)]. In both cases, at the 1.5 : 1 ratio, the pores were clear with sharp corners [Fig. 3(A and D)]. Whereas, at the 1 : 1 ratio, the droplets were corral shaped with some slight fractal characters that had open pores [Fig. 3(B and E)]. As the TMPTMP content increased, the fractal nanostructures with wide pore size distribution were seen clearly [Fig. 3(C and F)]. It can be said that these fractal structures arose when the amount of TMPTMP was greater than the TMPDE in the presence of an acrylate monomer (TEGDA). The fractal structures formation was reported earlier in thiol–acrylate^40^ and thiol–ene polymerizations.^35^ An artistic impression of fractal structures with a broad pore size distribution is shown in Fig. 3(G). The SEM images in Fig. 3(B and E) show interconnected pore clusters with large surface areas, which means that the LCs droplets sizes in the PDLCs with the highest Δ*T* are larger than the other PDLCs. When the fractal structured LCs droplet sizes were compared, the droplet size tended to increase as the TMPDE/TMPTMP ratio decreased. This may be attributed to the volume taken by the TMPTMP while it was connected to the polymer chains as a cross-linker.

### General switching mechanism in PDLCs

The PDLCs exhibited switching behavior, which led to the transparent (ON) and opaque (OFF) states when a suitable electric field was applied. The applied electric field aligned the LCs directors along the direction of the applied electric field that facilitated the easy passage of light with negligible scattering, which led to an ON state. When no electric field was applied, the light scattered due to different refractive indexes between the polymer and the LC with the randomly oriented directors, which in turn led to the OFF state. In the case of the dye based PDLCs, dye molecules with long chains tend to align parallel to the LCs. The orientation of LCs control the movement of the dye molecules, which led to the colorful PDLCs in the OFF state as dyes absorb the incident light.^23,30^ However, in the ON state, the dyes were parallel to the incident light and rarely absorbed it, so they became transparent. Some fainted color in the ON state was attributed to the dye molecules entrapped in the polymer matrix. The general schematic of the ON/OFF mechanism of PDLCs is shown in Fig. 3(H).

### Electro-optical studies

**Fig. 4:**
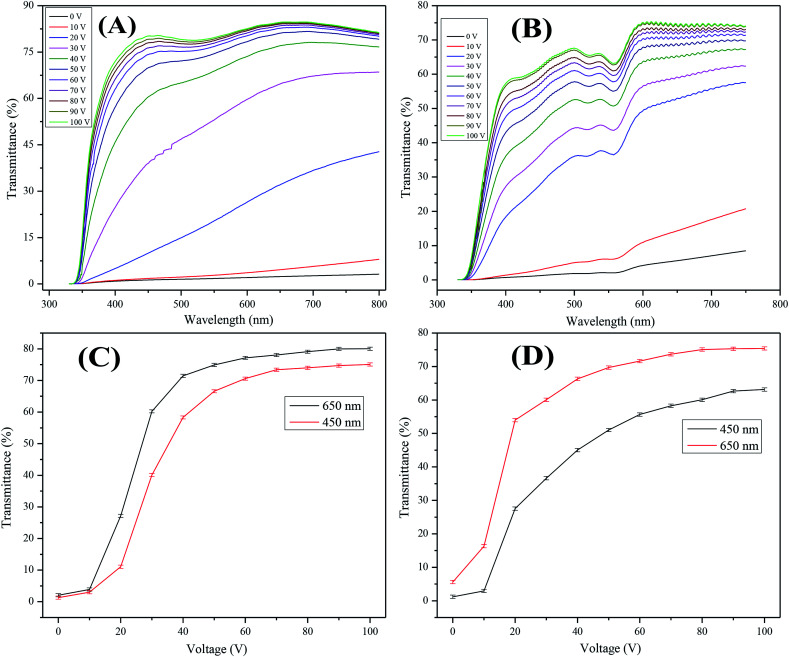
Transmittances *vs.* wavelength at various voltages are plotted for (A) non-dye PDLCs at 1 : 1 TMPDE/TMPTMP ratio and (B) red colored PDLCs at 1 : 1.25 TMPDE/TMPTMP ratio. (C and D) Transmittances *vs.* voltage are plotted at 450 and 650 nm wavelengths for non-dye and red colored PDLCs, respectively.

The field dependent transmittance was analyzed using UV-visible spectroscopy at different voltages (0–100 *V*_ac_). The voltage dependent transmittance spectra of the optimized non-dye and the colored PDLCs, which used air as reference, is shown in Fig. 4(A and B), respectively. It can be seen that transmittance enhances as the voltage is increased, which is a general behavior in PDLCs. It was estimated that Δ*T* was ∼77% and ∼75% for the non-dye and the colored PDLCs, respectively. The threshold voltage (*V*_th_) was slightly higher in the non-dye than that for the colored PDLCs, which is indicated in Fig. 4(C and D). The lower *V*_th_ in the colored PDLC can be explained by the wider droplet size distribution as shown in Fig. 3(F), because the LC in the larger droplets can be aligned at lower voltage. The non-dye and the colored PDLCs attained saturated transmittances at ∼70 and 80 V, respectively. The higher saturation voltage in the colored PDLC is related with the existence of dye in the LC droplet, because the stronger electric field at the LC droplet is required to rotate the dye molecules. In the colored PDLCs, there is a shallow valley between the 500–600 nm region in Fig. 4(B), which is ascribed to the absorbance of red colored dye molecules.

The transmittance variation trend at 450 and 650 nm wavelengths exhibited a continuous slope of transmittance with voltage increase. This behavior supported the formation of fractal structures with wide pore size distribution. The electrical force aligning LC and anchoring force from polymer matrix are in competition. While the electrical force is determined by the voltage, the anchoring force is inversely proportional to the distance from the interface. In general, if the size of the LCs droplets is small, the higher *V*_th_ is required to overcome stronger anchoring force, and if the droplet size is large, then the *V*_th_ is lower.^41–44^ When an intermediate voltage is applied, the LCs large droplets are aligned, but small droplets are not. The portion of the aligned droplets will be continuously changed depending on the voltage. This continuous switching behavior is advantageous for dimming.

### Switching response time (*τ*)

**Fig. 5:**
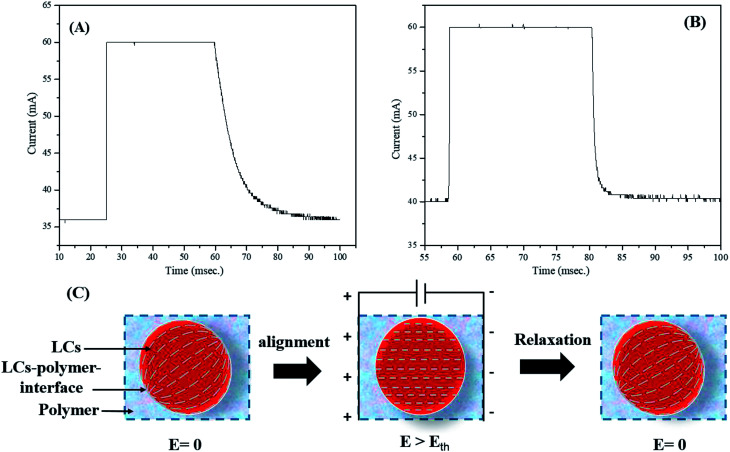
Switching time response for (A) non-dye PDLCs and (B) red colored PDLCs. (C) Alignment of LCs in droplets along the field and relaxation by the interfacial anchoring force.

Switching response time, which includes turn-on and turn-off times (*τ*_ON_ and *τ*_OFF_), was determined using a system that consisted of a laser, a photodiode, and an oscilloscope. The switching time response spectra of non-dye and red colored PDLCs are shown in Fig. 5(A and B). It was observed that the non-dye PDLCs had fast *τ*_ON_ ∼ 0.2 ms, whereas the red colored PDLCs had a slightly longer switching time of ∼0.3 ms. Both types of PDLCs had much longer turn-off times (*τ*_OFF_), such as ∼36 ms and ∼17 ms, respectively, than the turn-on times [Fig. 5(A and B)]. Actually, the switching response was affected by two forces as shown in Fig. 5(C). First, the electric field (*E*) aligned the LCs in an applied electric field direction during turning-on, and the interfacial anchoring force at the LCs–polymer boundary reoriented the alignment of the LCs along the tangential direction during turning-off. In general, *τ*_ON_ is dependent of the LCs droplets size. However, as the sizes of the droplets are not much different between the non-dye and colored PDLCs, there must be another mechanism determining *τ*_ON_. The longer *τ*_ON_ for the colored PDLC is supposed to be related with the existence of the dye. Because the dye did not respond to the electric field change directly, it slowed down the quick motion of the LC molecules. On the other hand, the long *τ*_OFF_ in both types of PDLCs may be ascribed to the polymer–LCs interface force.^44,45^ No electric field (*E* = 0) was applied during the relaxation process, and the relaxation into disordered state was based on the surface anchoring process.^46^ Because no energy was supplied and surface anchoring force was weaker than that from the external field, the *τ*_OFF_ was longer than the *τ*_ON_. In this process, the dye may have facilitated the relaxation of the LC to lower the free energy, because it had a different molecular structure from the LC. Hence, the colored PDLC exhibited a shorter *τ*_OFF_ than that of the non-dye PDLC by means of the dye.

### Ageing effect and power consumption in PDLCs

**Fig. 6:**
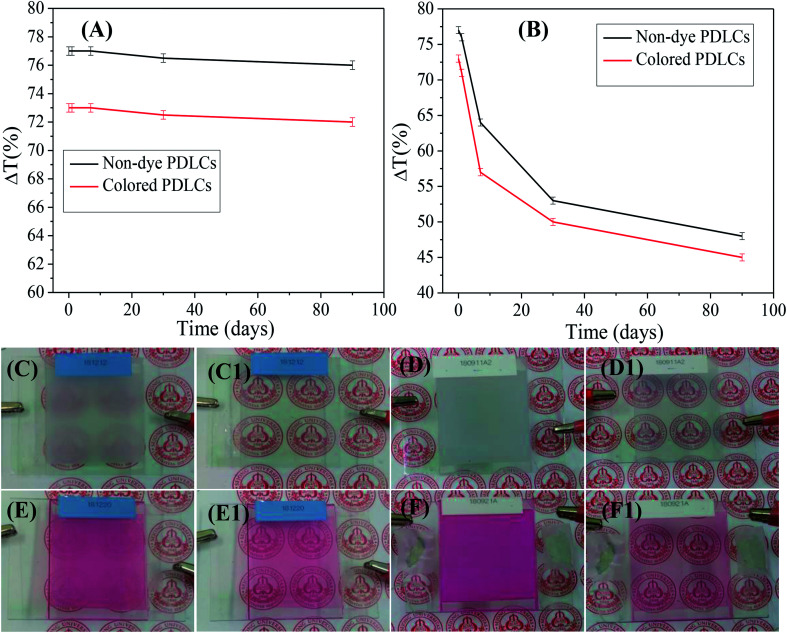
Ageing analysis of (A) liquid PDLC mixtures and (B) fabricated PDLC cells. Photographs are shown for the OFF/ON states of (C/C1) non-dye and (E/E1) red colored PDLCs fabricated with a 90 days-old mixture. The OFF/ON states for (D/D1) non-dye and (F/F1) colored-PDLC cells were inspected after 90 days since they were fabricated.

Ageing, such as the durability of PDLCs with time, is another important parameter in PDLCs fabrication. A good PDLCs cell must be sustainable for long time. Ageing has two aspects, which include the sustainability of liquid PDLCs mixture before UV curing and the sustainability of a fabricated UV-cured PDLCs cell. To analyze the ageing effect of the liquid PDLC mixture itself, it was mixed, and it became UV-cured in an ITO coated glass cell around three months later. The trend of Δ*T* is shown in Fig. 6(A) as a function of the ageing time, where the serious ageing effect is revealed. It was been found that the Δ*T* continuously decreased with time due to mixture ageing, and the decrement in Δ*T* was found to be ∼35–40% in both non-dye and colored PDLCs. The corresponding visual images of PDLCs fabricated with a 90 days-old mixture are shown in Fig. 6(C/C1 and E/E1). The OFF-states became slightly transparent, and the ON-states were not completely opaque due to the degradation of the mixture. It was estimated that the PDLCs mixture was sustainable only up to 24 hours. On the other hand, the PDLC cells were stable once they were cured, and Δ*T* reduced marginally by ∼1% in non-dye and red colored PDLCs in 90 days. Fig. 6(D/D1 and F/F1) show the ON/OFF states of PDLCs after 90 days. The deterioration in the non-cured mixture was obviously due to a chemical reaction between the constituents, whereas the stability of the UV-cured PDLCs cells was due to curing of the mixed constituents with UV-light, which removed the possibility of further chemical reaction after the click reaction.

The power consumption is an important parameter to decide the commercial viability of PDLCs in smart windows. The power of PDLCs was estimated by connecting the PDLCs in series with a multimeter and AC power supply. From the measured voltage and current, the power was determined, which was divided by area of the PDLCs cell (W m^−2^). The power consumption was observed as low as ∼6 W m^−2^ and ∼6.4 W m^−2^ in the cases of non-dye and colored PDLCs, respectively. The power consumption of colored PDLCs slightly higher than non-colored PDLCs may be ascribed to additional force required to align the LCs molecules with dye molecules together. It also corroborates the longer turn ON time in colored PDLCs. These power consumptions are negligible compared with the expected power saving when it is applied as a window, considering the solar radiation of ∼1000 W m^−2^, typically.

## Conclusions

Colored PDLCs have been fabricated using acrylate and thiol–ene monomers with just ∼55 wt% LCs content. Various optimizations have been made to ensure the best monomers ratio combination to obtain higher Δ*T*, and other performance parameters in non-colored and colored PDLCs. LCs/TEGDA have been optimized best at a 2 : 1 ratio. The best optimized ratio of TMPDE/TMPTMP (at LCs/TEGDA = 2 : 1) was found as 1 : 1 in non-dye PDLCs, whereas as in colored PDLCs it was 1 : 1.25. The colors have been introduced by adding red and blue dichroic dyes. In the case of TEGDA/TMPDE/TMPTMP based PDLCs, the colors are not changed after UV-curing, but in the case of NOA65, the colors changed to a yellowish color. The LCs droplets exhibited fractal-structured geometry, and the fractalization character enhanced with increased TMPTMP content. The LCs droplet size distribution was wider in the case of the colored PDLCs. The colored PDLCs had Δ*T* >70% at relatively low voltage ∼70 V, a fast switching time as low as ∼17 ms with low power consumption < 7 W m^−2^. The PDLCs mixtures are prone to be degraded with time, whereas the UV-cured PDLCs, with and without dye, were found stable with negligible change in Δ*T* even after 90 days. This acrylate-assisted thiol–ene combination at relatively low LCs content, with high Δ*T*, low power consumption, and good switching time may offer a myriad of opportunities in commercial applications, particularly in aesthetic vibrant colored energy-efficient smart windows.

## Conflicts of interest

There are no conflicts to declare.

## Supplementary Material

RA-009-C9RA00729F-s001

## References

[cit1] Long L. S., Ye H. (2014). How to be smart and energy efficient: a general discussion on thermochromic windows. Sci. Rep..

[cit2] Ghosh A., Mallick T. K. (2018). Evaluation of optical properties and protection factors of a PDLC switchable glazing for low energy building integration. Sol. Energy Mater. Sol. Cells.

[cit3] Liang X., Chen M., Wang Q., Guo S. M., Zhang L. Y., Yang H. (2018). Active and passive modulation of solar light transmittance in a hybrid thermochromic soft-matter system for energy-saving smart window applications. J. Mater. Chem. C.

[cit4] Liang X., Guo S. M., Chen M., Li C. Y., Wang Q., Zou C., Zhang C. H., Zhang L. Y., Guo S. J., Yang H. (2017). A temperature and electric field-responsive flexible smart film with full broadband optical modulation. Mater. Horiz..

[cit5] Gu Y., Hong W., Choi W., Park J. Y., Kim K. B., Lee N., Seo Y. (2014). Electrochromic Device Containing Heptyl Viologen, PEDOT, TiO_2_ and TEMPO. J. Electrochem. Soc..

[cit6] Ghosh A., Norton B., Duffy A. (2016). Daylighting performance and glare calculation of a suspended particle device switchable glazing. Sol. Energy.

[cit7] Ghosh A., Norton B., Duffy A. (2016). Behaviour of a SPD switchable glazing in an outdoor test cell with heat removal under varying weather conditions. Appl. Energy.

[cit8] Natarajan L. V., Shepherd C. K., Brandelik D. M., Sutherland R. L., Chandra S., Tondiglia V. P., Tomlin D., Bunning T. J. (2003). Switchable holographic polymer-dispersed liquid crystal reflection gratings based on thiol-ene photopolymerization. Chem. Mater..

[cit9] Zhou L., Ma H. P., Han C., Hu W., Zhang S. F., Zhang L. Y., Yang H. (2018). A novel light diffuser based on the combined morphology of polymer networks and polymer balls in a polymer dispersed liquid crystals film. RSC Adv..

[cit10] Liu Y. J., Ding X. Y., Lin S. C. S., Shi J. J., Chiang I. K., Huang T. J. (2011). Surface Acoustic Wave Driven Light Shutters Using Polymer-Dispersed Liquid Crystals. Adv. Mater..

[cit11] Sheraw C. D., Zhou L., Huang J. R., Gundlach D. J., Jackson T. N., Kane M. G., Hill I. G., Hammond M. S., Campi J., Greening B. K., Francl J., West J. (2002). Organic thin-film transistor-driven polymer-dispersed liquid crystal displays on flexible polymeric substrates. Appl. Phys. Lett..

[cit12] Nicoletta F. P., Chidichimo G., Cupelli D., De Filpo G., De Benedittis M., Gabriele B., Salerno G., Fazio A. (2005). Electrochromic polymer-dispersed liquid-crystal film: a new bifunctional device. Adv. Funct. Mater..

[cit13] Hoyle C. E., Bowman C. N. (2010). Thiol-Ene Click Chemistry. Angew. Chem., Int. Ed..

[cit14] Shi Z. Q., Shao L. S., Wang F., Deng F. F., Liu Y. W., Wang Y. H. (2018). Fabrication of dye-doped polymer-dispersed liquid crystals with low driving voltage based on nucleophile-initiated thiol-ene click reaction. Liq. Cryst..

[cit15] Cramer N. B., Scott J. P., Bowman C. N. (2002). Photopolymerizations of thiol-ene polymers without photoinitiators. Macromolecules.

[cit16] Zhang Y., Zhou L., Yang J., Zhang J., Hai M., Zhang L., Li F., Zhang C., Yang Z., Yang H., Zhu S. (2018). Effects of crosslinking agent/diluents/thiol on morphology of the polymer matrix and electro-optical properties of polymer-dispersed liquid crystal. Liq. Cryst..

[cit17] Shi Z. Q., Shao L. S., Zhang Y. L., Guan Y., Wang F., Deng F. F., Liu Y. W., Wang Y. H. (2017). Fabrication of polymer-dispersed liquid crystals with low driving voltage based on the thiol-ene click reaction. Polym. Int..

[cit18] Yaroshchuk O., Elouali F., Maschke U. (2010). Control of phase separation and morphology of thiol-ene based PDLCs by curing light intensity. Opt. Mater..

[cit19] Park S., Kim H. K., Hong J. W. (2010). Investigation of the photopolymerization-induced phase separation process in polymer dispersed liquid crystal. Polym. Test..

[cit20] Lee J. W., Kim J. K., Ahmad F., Jamil M., Jeon Y. J. (2014). Properties of thiol-vinyl PDLC films without additional photoinitiator. Liq. Cryst..

[cit21] Cho J. D., Lee S. S., Park S. C., Kim Y. B., Hong J. W. (2013). Optimization of LC droplet size and electro-optical properties of acrylate-based polymer-dispersed liquid crystal by controlling photocure rate. J. Appl. Polym. Sci..

[cit22] Shi Z., Shao L., Wang F., Deng F., Liu Y., Wang Y. (2018). Fabrication of dye-doped polymer-dispersed liquid crystals with low driving voltage based on nucleophile-initiated thiol-ene click reaction. Liq. Cryst..

[cit23] Lee S. H., Lim T. K., Shin S. T., Park K. S. (2002). A method for improving contrast ratio of polymer dispersed liquid crystal film using the oriented azo-dye molecules in polymer matrix. Jpn. J. Appl. Phys., Part 1.

[cit24] Kim M., Park K. J., Seok S., Ok J. M., Jung H. T., Choe J., Kim D. H. (2015). Fabrication of Microcapsules for Dye-Doped Polymer-Dispersed Liquid Crystal-Based Smart Windows. ACS Appl. Mater. Interfaces.

[cit25] Malik P., Raina K. K. (2010). Dichroic dye-dependent studies in guest-host polymer-dispersed liquid crystal films. Phys. B.

[cit26] Oh S. W., Baek J. M., Heo J., Yoon T. H. (2016). Dye-doped cholesteric liquid crystal light shutter with a polymer-dispersed liquid crystal film. Dyes Pigm..

[cit27] Liu Y. J., Sun X. W., Elim H. I., Ji W. (2006). Gain narrowing and random lasing from dye-doped polymer-dispersed liquid crystals with nanoscale liquid crystal droplets. Appl. Phys. Lett..

[cit28] West J. L., Ondriscrawford R. (1991). Characterization of Polymer Dispersed Liquid-Crystal Shutters by Ultraviolet Visible and Infrared-Absorption Spectroscopy. J. Appl. Phys..

[cit29] González-Vargas C., Salazar R., Sirés I. (2014). Electrochemical treatment of Acid Red 1 by electro-Fenton and photoelectro-Fenton processes. J. Electrochem. Sci. Eng..

[cit30] Chen Z. H., Swager T. M. (2007). Synthesis and characterization of fluorescent acenequinones as dyes for guest-host liquid crystal displays. Org. Lett..

[cit31] Lowe A. B. (2014). Thiol-ene “click” reactions and recent applications in polymer and materials synthesis: a first update. Polym. Chem..

[cit32] Deshmukh R., Malik M. (2013). Photopolymerisation kinetics and electro-optical properties in mixtures of dichroic dye-doped nematic liquid crystal and photocurable polymer. Liq. Cryst..

[cit33] Ahmad F., Jamil M., Jeon Y. J., Woo L. J., Jung J. E., Jang J. E. (2012). Investigation of nonionic diazo dye-doped polymer dispersed liquid crystal film. Bull. Mater. Sci..

[cit34] White T. J., Natarajan L. V., Tondiglia V. P., Bunning T. J., Guymon C. A. (2007). Polymerization kinetics and monomer functionality effects in thiol-ene polymer dispersed liquid crystals. Macromolecules.

[cit35] Chiou B. S., English R. J., Khan S. A. (1996). Rheology and photo-cross-linking of thiol-ene polymers. Macromolecules.

[cit36] Langford C. R., Johnson D. W., Cameron N. R. (2014). Chemical functionalization of emulsion-templated porous polymers by thiol-ene "click" chemistry. Polym. Chem..

[cit37] Cramer N. B., Reddy S. K., Cole M., Hoyle C., Bowman C. N. (2004). Initiation and kinetics of thiol-ene photopolymerizations without photoinitiators. J. Polym. Sci., Part A: Polym. Chem..

[cit38] Sahin M., Ayalur-Karunakaran S., Manhart J., Wolfahrt M., Kern W., Schlogl S. (2017). Thiol-Ene *versus* Binary Thiol-Acrylate Chemistry: Material Properties and Network Characteristics of Photopolymers. Adv. Eng. Mater..

[cit39] Lee T. Y., Carioscia J., Smith Z., Bowman C. N. (2007). Thiol-allyl ether-methacrylate ternary systems. Evolution mechanism of polymerization-induced shrinkage stress and mechanical properties. Macromolecules.

[cit40] Higham A. K., Garber L. A., Latshaw D. C., Hall C. K., Pojman J. A., Khan S. A. (2014). Gelation and Cross-Linking in Multifunctional Thiol and Multifunctional Acrylate Systems Involving an in Situ Comonomer Catalyst. Macromolecules.

[cit41] Peng S. C., Yu J. W., Lee S. N. (1997). Effect of droplet size on the dielectric properties of PDLC films. J. Polym. Sci., Part B: Polym. Phys..

[cit42] Li W. B., Cao Y. B., Cao H., Kashima M., Kong L. J., Yang H. (2008). Effects of the structures of polymerizable monomers on the electro-optical properties of UV cured polymer dispersed liquid crystal films. J. Polym. Sci., Part B: Polym. Phys..

[cit43] Maschke U., Coqueret X., Benmouna M. (2002). Electro-optical properties of polymer-dispersed liquid crystals. Macromol. Rapid Commun..

[cit44] Zhang T. T., Kashima M., Zhang M. Z., Liu F., Song P., Zhao X. T., Zhang C. H., Cao H., Yang H. (2012). Effects of the functionality of epoxy monomer on the electro-optical properties of thermally-cured polymer dispersed liquid crystal films. RSC Adv..

[cit45] Jung D., Choi W., Park J. Y., Kim K. B., Lee N., Seo Y., Kim H. S., Kong N. K. (2017). Inorganic gel and liquid crystal based smart window using silica sol-gel process. Sol. Energy Mater. Sol. Cells.

[cit46] Kim Y., Jung D., Jeong S., Kim K., Choi W., Seo Y. (2015). Optical properties and optimized conditions for polymer dispersed liquid crystal containing UV curable polymer and nematic liquid crystal. Curr. Appl. Phys..

