# Supplementary material for: Acrylate-assisted fractal nanostructured polymer dispersed liquid crystal droplet based vibrant colored smart-windows
Source: RSC Adv. 2019 Apr 24;9(22):12645–55. doi: 10.1039/c9ra00729f (PMC9063710; doi:10.1039/c9ra00729f)
Supplement: RA-009-C9RA00729F-s001 [file RA-009-C9RA00729F-s001.pdf]

Electronic Supplementary Material (ESI) for RSC Advances.
This journal is © The Royal Society of Chemistry 2019

Supporting information

Acrylate-assisted Fractal Nanostructured Polymer Dispersed Liquid Crystals Droplets Based Vibrant Colored Smart-Windows

Sunil Kumar[1,2], Hyeryeon Hong[2], Woosuk Choi[2], Imtisal Akhtar[2], Malik Abdul Rehman[2], Yongho Seo[1,2*]

[1]Graphene Research Institute and HMC, Sejong University, Seoul, 05006, South Korea

[2]Department of Nanotechnology and Advanced Materials Engineering, Sejong University, Seoul, 05006, South Korea

**corresponding author: yseo@sejong.ac.kr*

1. ΔT for different PDLCs compositions

PDLCs were fabricated with the LCs (~55wt.%) and TEGDA (45wt.%) only, another PDLCs fabricated using LCs (55wt.%) with TMPDE (22.5wt.%) and the TMPTMP (22.5wt.%) to check the effect of the TEGDA with TMPDE and the TMPTMP combination. Also, LCs (55wt.%) NOA65 (~45wt.%) with and without red and blue dyes (both 0.7wt.%, separately) based PDLCs were fabricated for a color comparison at similar LCs, monomers, and dyes concentrations. The associated parameters are shown in Table S1, and the corresponding colors of PDLCs are shown in Figure S2.

Table S1. ΔT (%) for different PDLCs compositions. The numbers in brackets are concentration in wt.%

S. No.	PDLCs composition (concentration, wt.%)	ΔT (%)	Remark
1.	LCs (55) + TEGDA (45)	31	Low ΔT
2.	LCs (55) + TEMPDE (22.5) + TMPTMP (22.5)	35	Low ΔT
3.	LCs (55) +NOA65 (45)	67	Low ΔT
4.	LCs (54.3) + NOA65 (45) + red dye AR1 (0.7)	61	Color changes to yellow
5.	LCs (54.3) + NOA65 (45) + blue dye AB4(0.7%)	54	Color changes to yellow
6.	LCs (54.3) + TEGDA (27) + TEMPDE (8) + TMPTMP (10) + blue dye AB4 (0.7)	60	Color remains blue

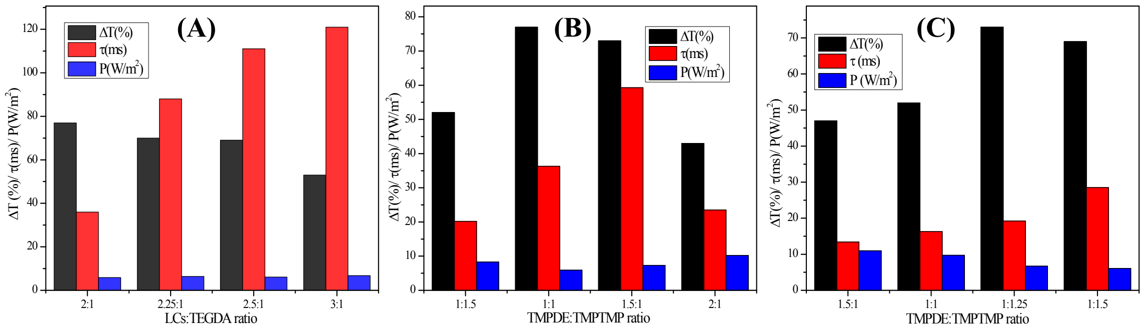

Figure S1. Variation of ΔT (%), power (P) and switching time (τ) at (A) different LCs/TEGDA ratio, (B) non-dye PDLCs, and (C) red colored PDLCs

2. Photographs in OFF/ON states of various PDLCs

LCs with TEGDA only (Figure S2 (A-A1)), and LCs with TMPDE and TMPTMP based PDLCs (Figure S2 (B-B1)) have low ΔT, ~31% and 35%, respectively. In the case of NOA65 based PDLCs with non-dye, red, and blue dye based PDLCs (Figure S2 (C-E1)), it was ~67%, ~61%, and ~54% respectively, under similar conditions. After UV-curing the color of NOA65 based PDLCs with the blue or red dye was changed to a yellowish color with reduced transmittance (Figure S2 (D-E)). However, in the case of TEGDA/TMPDE/TMPTMP based PDLCs with blue dye, the color remains the same and ΔT(%) ~60% (Figure S2 (F/F1)). The color change in NOA65 based PDLCs may be due to the fact that NOA65 has photoinitiator like benzophenone and isophorone diisocyanate (IPDI) additionally, whereas the other PDLCs have no photoinitiator.

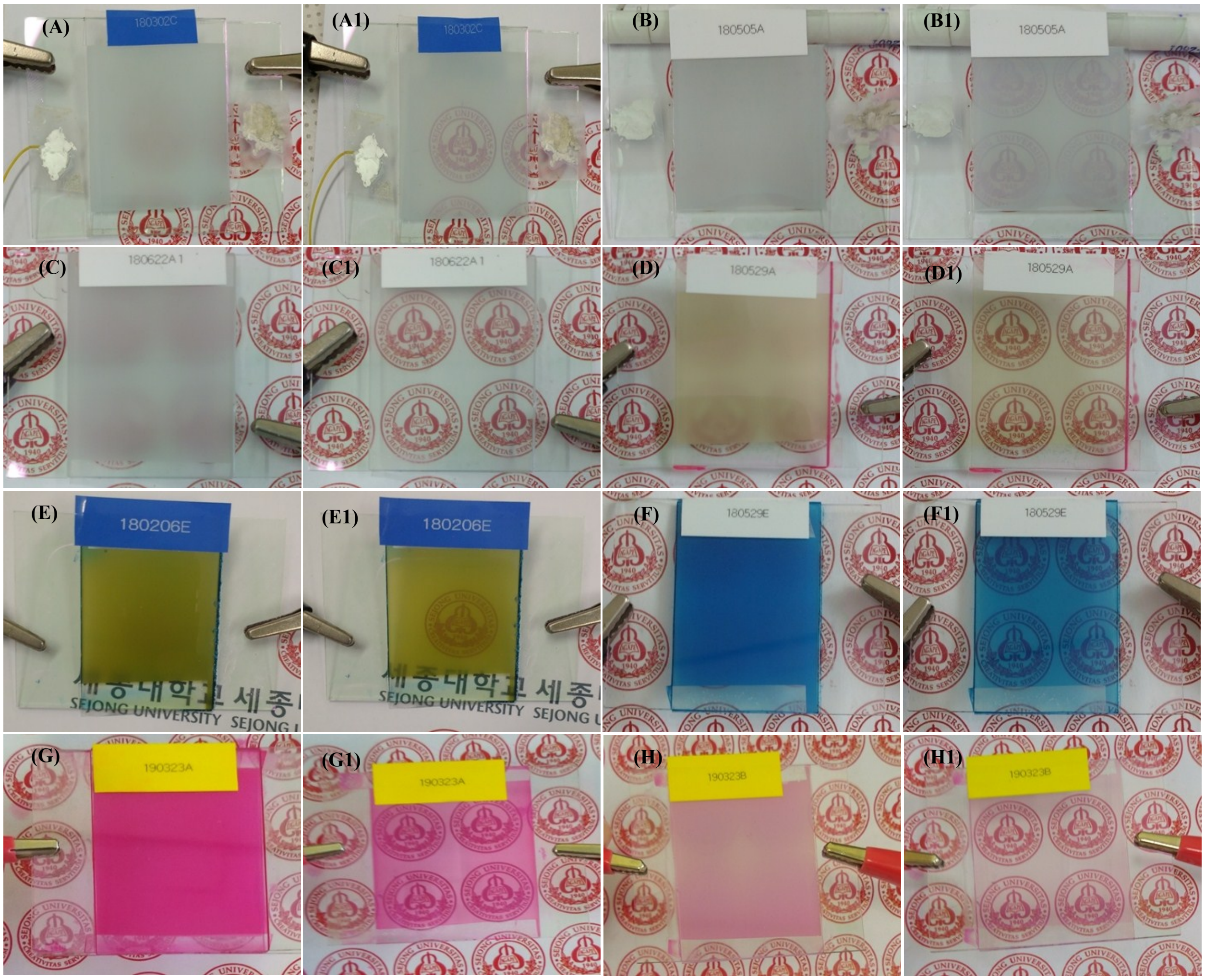

Figure S2. Photographs in OFF/ON states of various PDLCs: (A/A1) LCs+ TEGDA, (B/B1) LCs+ TMPDE+ TMPTMP, (C/C1) LCs+ NOA65, (D/D1) LCs+ NOA65 + red AR1 dye, (E/E1) LCs+ NOA65+ blue AB4 dye, (F/F1) LCs+ TEGDA+ TMPDE+ TMPTMP+ blue AB4 dye, (G/G1) LCs+ TEGDA+ TMPDE+ TMPTMP+ 1wt.% red AR1 dye, and (H/H1) LCs+ TEGDA+ TMPDE+ TMPTMP+ 0.5wt.% red AR1 dye based PDLCs.

3. Chemical formulas for PDLC ingredients

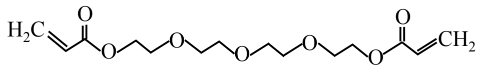

TEGDA

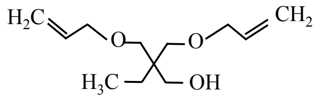

TMPDE

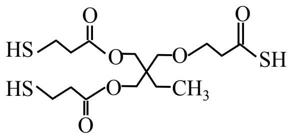

TMPTMP

Figure S3. Chemical formulas of TEGDA, TMPDE, and TMPTMP. TEGDA and TMPDE have two double C=C bonds.
